# Commentary: Aripiprazole disrupts cellular synchrony in the suprachiasmatic nucleus and enhances entrainment to environmental light–dark cycles in mice

**DOI:** 10.3389/fnins.2024.1371195

**Published:** 2024-04-19

**Authors:** Tsuyoshi Kitajima

**Affiliations:** Department of Psychiatry, Fujita Health University School of Medicine, Toyoake, Japan

**Keywords:** aripiprazole, circadian rhythm sleep-wake disorders, delayed sleep-wake phase disorder, suprachiasmatic nucleus, sleep-wake cycle

## 1 Introduction

Among circadian rhythm sleep–wake disorders (CRSWDs), delayed sleep–wake phase disorder (DSWPD) is particularly disabling for the youth. It has been reported that aripiprazole, a second-generation antipsychotic, advances the sleep phase in DSWPD (Takaki and Ujike, 2014; Tashiro, 2017; Omori et al., 2018; Konishi et al., 2022). Although this needs to be supported by further evidence, it may be a potent therapeutic option. In a previous issue of Frontiers in Neuroscience, Li et al. (2023) reported that aripiprazole swiftly reset locomotor rhythm of mice following an acute shift in the light–dark cycle. Additionally, they demonstrated *ex vivo* that aripiprazole loosened the synchronization of cellular oscillations in mice suprachiasmatic nucleus (SCN) slices via 5-HT_1A_R action, leading to a reduction in the amplitude of SCN oscillation. Moreover, the authors discussed that its resetting action on mice's locomotor rhythm may be the central origin, and this scheme might also be applied to human DSWPD.

The findings of this study seem quite valuable, providing new insight into effects of aripiprazole on the circadian regulatory system. The concept that the damping of SCN oscillation amplitude may bring flexibility to circadian adaptation has been claimed by another mouse study (Noguchi et al., 2020). Moreover, there has also been an argument that people with lower circadian amplitude may have better adaptation to night-shift work (Radosević-Vidacek et al., 1993). However, we need to further consider whether the aripiprazole's action on human DSWPD can be attributable to change of the SCN function, as well as the possibility of its direct action on sleep-wake cycle in both of mice and humans.

## 2 Mechanism of resetting the mice circadian rhythm by aripiprazole

Li et al. demonstrated separately that aripiprazole reset the locomotor rhythm of mice, and dampened the oscillation amplitude in mice SCN; thus, they did not directly prove that the results *in vivo* were attributable to functional change in the SCN. Honma and Honma (2009) demonstrated that methamphetamine, a dopaminergic stimulant, drives the sleep–wake cycle independent of the SCN in rats. As aripiprazole is a dopamine partial agonist, the possibility that this agent directly modulates the sleep–wake cycle of mice needs to be discussed.

## 3 Application of the results of this study to human CRSWDs

Aripiprazole dosage used in this study was much higher in both *in vivo* and *ex vivo* experiments than that in human clinical usage. However, the reported dose required for human DSWPD is 0.5–3 mg a day (Omori et al., 2018), which is much lower than that for human schizophrenia or bipolar disorder (6–30 mg a day). The *ex vivo* experiments in this study revealed that aripiprazole did not dampen the amplitude of SCN at a relatively low dose (Li et al., 2023). Thus, it is unclear whether such a low dose of aripiprazole for DSWPD could modulate the oscillation of the SCN in humans. If this were true, a relatively higher dose of aripiprazole would have been more effective for DSWPD.

Another point would be how the dampened amplitude of the SCN oscillation can promote the sleep-wake's adaptation to the light-dark cycle in human CRSWDs. If the phase of the SCN rhythm itself becomes more flexible to shift, the mechanism should be “top-down,” as discussed below. If the SCN loses the sleep-wake cycle regulation and lets the cycle shift rapidly, this may also make it more irregular, like in patients with SCN lesions (Attarian, 2009; DelRosso et al., 2014).

## 4 Discussion

If aripiprazole has efficacy for DSWPD, there might be alternative mechanisms other than the direct effect on the SCN. One of them is that it might promote earlier awakening in the morning, possibly by dopamine partial agonism, and the patient can be exposed to light at that time. One of the hypotheses of DSWPD etiology is that a later sleep phase would mask the phase advance zone of the phase response curve for light (Uchiyama et al., 2000a). Aripiprazole might uncover the critical time zone for light and advance the phase of the central pacemaker. Another explanation is that aripiprazole modulates the sleep–wake cycle independently of the central pacemaker. There is another hypothesis of DSWPD etiology that its patients have a slow build-up of sleep homeostasis, leading to a delayed sleep–wake cycle (Uchiyama et al., 2000b; American Academy of Sleep Medicine, 2023). A similar mechanism has been proposed for depression (Borbély et al., 2016), and interestingly, aripiprazole is also effective for depression at a relatively low dose. Thus, aripiprazole might enhance the building up of sleep homeostasis and expedite the sleep–wake cycle. The last one might be behavioral modulation; for example, aripiprazole might stabilize impulsivity that might exacerbate bedtime procrastination, specifically in people with a tendency of attention deficit. These “bottom-up” mechanisms might account for the action of aripiprazole on DSWPD rather than the modulation of the “top–down” circadian control.

Recent studies have reported that over 40% of DSWPD patients do not have a delayed melatonin rhythm (Duffy et al., 2021; American Academy of Sleep Medicine, 2023). If aripiprazole has “top–down” action for the human circadian system, it would be more effective for patients with delayed melatonin rhythm, or if its action is “bottom–up,” this would be the opposite. This should be investigated in future studies. Similarly, it is claimed that non-24-h sleep–wake rhythm disorder (N24SWD) with a far longer sleep–wake cycle period (≥30 h) than the range of human intrinsic circadian period is not considered to be primarily driven by the central circadian pacemaker (Emens et al., 2022). And it is notable that aripiprazole was reported to be effective in a case with N24SWD and major depressive disorder, whose sleep–wake cycle period was 33.4 h (Matsui et al., 2017).

Thus, we should carefully discuss whether the action of aripiprazole on human circadian rhythm is directly related to the SCN. Further studies are needed to address this, and these may also contribute to further elucidating the etiology of CRSWDs.

## Author contributions

TK: Writing—original draft, Writing—review & editing.
